# The effectiveness of evidence summaries on health policymakers and health system managers use of evidence from systematic reviews: a systematic review

**DOI:** 10.1186/s13012-016-0530-3

**Published:** 2016-12-09

**Authors:** Jennifer Petkovic, Vivian Welch, Maria Helena Jacob, Manosila Yoganathan, Ana Patricia Ayala, Heather Cunningham, Peter Tugwell

**Affiliations:** 1 University of Split School of Medicine, Split, Croatia; 2 Bruyère Research Institute, University of Ottawa, 43 Bruyère Street, Annex E room 302, Ottawa, ON K1N 5C8 Canada; 3 School of Epidemiology, Public Health and Preventive Medicine, University of Ottawa, Ottawa, Canada; 4 Gerstein Science Information Centre, University of Toronto, Toronto, Canada; 5 Department of Medicine, Faculty of Medicine, University of Ottawa, Ottawa, Canada; 6 Ottawa Hospital Research Institute, Clinical Epidemiology Program, Ottawa, Canada; 7 Department of Epidemiology and Community Medicine, Faculty of Medicine, University of Ottawa, Ottawa, Canada

**Keywords:** Systematic reviews, Policymakers, Evidence summaries

## Abstract

**Background:**

Systematic reviews are important for decision makers. They offer many potential benefits but are often written in technical language, are too long, and do not contain contextual details which make them hard to use for decision-making. There are many organizations that develop and disseminate derivative products, such as evidence summaries, from systematic reviews for different populations or subsets of decision makers. This systematic review aimed to (1) assess the effectiveness of evidence summaries on policymakers’ use of the evidence and (2) identify the most effective summary components for increasing policymakers’ use of the evidence. We present an overview of the available evidence on systematic review derivative products.

**Methods:**

We included studies of policymakers at all levels as well as health system managers. We included studies examining any type of “evidence summary,” “policy brief,” or other products derived from systematic reviews that presented evidence in a summarized form. The primary outcomes were the (1) use of systematic review summaries in decision-making (e.g., self-reported use of the evidence in policymaking and decision-making) and (2) policymakers’ understanding, knowledge, and/or beliefs (e.g., changes in knowledge scores about the topic included in the summary). We also assessed perceived relevance, credibility, usefulness, understandability, and desirability (e.g., format) of the summaries.

**Results:**

Our database search combined with our gray literature search yielded 10,113 references after removal of duplicates. From these, 54 were reviewed in full text, and we included six studies (reported in seven papers) as well as protocols from two ongoing studies. Two studies assessed the use of evidence summaries in decision-making and found little to no difference in effect. There was also little to no difference in effect for knowledge, understanding or beliefs (four studies), and perceived usefulness or usability (three studies). Summary of findings tables and graded entry summaries were perceived as slightly easier to understand compared to complete systematic reviews. Two studies assessed formatting changes and found that for summary of findings tables, certain elements, such as reporting study event rates and absolute differences, were preferred as well as avoiding the use of footnotes.

**Conclusions:**

Evidence summaries are likely easier to understand than complete systematic reviews. However, their ability to increase the use of systematic review evidence in policymaking is unclear.

**Trial registration:**

The protocol was published in the journal Systematic Reviews (2015;4:122)

**Electronic supplementary material:**

The online version of this article (doi:10.1186/s13012-016-0530-3) contains supplementary material, which is available to authorized users.

## Background

Policymakers are increasingly utilizing systematic reviews for decision-making [1–4]. The shift from single studies has occurred because systematic reviews offer additional benefits to policymakers, such as having lower risk of bias than other studies and offering more confidence in results than single studies [2]. However, since systematic reviews are often written using technical language, lack important contextual information, and can be quite long, research groups and organizations have begun creating summaries of the evidence [4, 5]. A needs assessment conducted by Evidence Aid found that while complete systematic reviews were perceived to be useful for workers “on the ground” (i.e., NGOs, health care providers), summaries containing contextual information were considered helpful for decision-making about the applicability of the findings to their local setting [6].

There are several organizations that develop and disseminate evidence summaries for different populations or subsets of decision makers. For example, within the Cochrane Collaboration, the Evidence Aid Project was developed in response to the 2004 Indian Ocean Tsunami as a means of providing decision makers and health practitioners on the ground with summaries of the best available evidence needed to respond to emergencies and natural disasters [6].

SUPPORT summaries were developed for policymakers in low- and middle-income countries (LMICs) making decisions about maternal and child health programs and interventions (http://supportsummaries.org/). Health Systems Evidence provides a one-stop shop for systematic reviews related to health systems including policy briefs for policymakers and other stakeholders (www.healthsystemsevidence.org/). Other examples include Cochrane Summaries (http://www.cochrane.org/evidence), Communicate to vaccinate (COMMVAC) (http://www.commvac.com), and Rx for change (https://www.cadth.ca/rx-change). A document analysis conducted by Adam et al. identified 16 organizations involved in the production of summaries for policymakers in LMICs [7]. These summaries are identified using many different terms including evidence summaries, policy briefs, briefing papers, briefing notes, evidence briefs, abstracts, summary of findings, and plain language summaries [7] but often contain summarized evidence from systematic reviews. They are intended to assist decision makers in understanding the evidence and encourage its use in their decision-making. These user-friendly formats highlight the policy-relevant information and allow policymakers to quickly scan the document for relevance [2, 8]. The various products have some differences. For example, abstracts, evidence summaries, and summary of findings tables summarize evidence from a single systematic review while policy briefs utilize evidence from one or more systematic reviews and may use additional sources to provide contextual or economic information [7].

Evidence on the usefulness and effectiveness of systematic review derivatives is lacking. Previously conducted systematic reviews have looked at interventions to increase the use of systematic reviews among decision makers; however, these have focused on the use of complete systematic reviews in decision-making, and none focused specifically on derivatives of systematic reviews. For example, one systematic review examined the effectiveness of interventions for improving the use of systematic reviews in decision-making by health system managers, policymakers, and clinicians [9]. This review included eight studies, and the authors concluded that information provided as a single, clear message may improve evidence-based practice, but increasing awareness and knowledge of systematic review evidence might require a multi-faceted intervention. Similarly, another systematic review assessed interventions encouraging the use of systematic reviews by health policymakers and managers [10]. Four studies were included, and the authors concluded that future research should identify how systematic reviews are accessed and the formats used to present the information. A systematic review by Wallace et al. found that the barriers, facilitators, and interventions that impact systematic review uptake found that a description of benefits as well as harms and costs and the use of a graded entry approach (in which evidence is available as a one-page summary, three-page summary, or 25-page full report) facilitated systematic review use by policymakers [11]. Similarly, a systematic review by Oliver et al. also assessed barriers and facilitators to the use of research by policymakers; they found that access to high-quality, relevant research as well as collaboration between researchers and policymakers were the most important factors for increasing research use [12].

This review aimed to assess the effectiveness of systematic review summaries on increasing policymakers’ use of systematic review evidence and to identify the components or features of these summaries that are most effective [13]. We present an overview of the available evidence on systematic review derivative products.

### Objectives

The objectives of this review were to (1) assess the effectiveness of evidence summaries on policy-makers’ use of the evidence and (2) identify the most effective summary components for increasing policy-makers’ use of the evidence.

## Methods

### Review protocol

We developed and published an a priori protocol for this systematic review [13].

### Searches

Information specialists (APA, HC) developed and translated the search strategy using the PRESS Guideline [14].

#### Electronic searches

We used the search strategy developed by Perrier et al. and Murthy et al. for their systematic reviews of interventions to encourage the use of systematic reviews by health managers and policymakers to inform our search [9, 10]. We expanded the Perrier search by including additional databases, as suggested by John Eyres, of the International Initiative for Impact Evaluation (3ie) and the Campbell International Development Review Group. These included Global Health Library (from WHO), Popline, Africa-wide, Public Affairs Information Service, Worldwide Political Science Abstracts, Web of Science, and DfiD (Research for Development Database). The search strategies were translated using each database platform’s command language and appropriate search fields. Both controlled vocabulary terms and text words were used for the search concepts of policymaking, evidence synthesis, systematic reviews, knowledge translation, and dissemination. No date restrictions were used. The complete MEDLINE search strategy is available in Additional file 1.

#### Searching other resources

We identified and searched websites of research groups and organizations which produce evidence summaries building on the list of organizations identified by Adam et al. [7]. We searched for unpublished studies evaluating the effectiveness of the systematic review derivatives in increasing policymakers’ understanding (e.g., Health Systems Evidence, the Canadian Agency For Drugs And Technologies In Health, SUPPORT summaries). A complete list of gray literature sources is provided in Additional file 2.

**Fig. 1:**
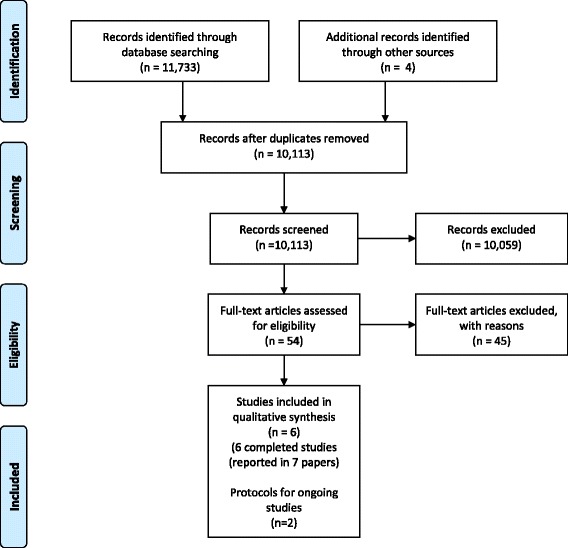
PRISMA flow diagram

We also checked the reference lists of included studies and related systematic reviews to identify additional studies. We contacted researchers to identify ongoing and completed/published work. The results of the search are reported in Fig. 1.

### Study inclusion and exclusion criteria

Eligible studies included randomised controlled trials (RCTs), non-randomised controlled trials (NRCTs), controlled before-after (CBA) studies, and interrupted time series (ITS) studies.

We included studies whose participants were health policymakers at all levels. We defined policymakers as health ministers and their political staff, civil servants, health system managers, and health system stakeholders as civil society groups, patient groups, professional associations, non-governmental organizations, donors, and international agencies [15]. We included populations involved in the development of clinical practice guidelines. To be included, the population had to be responsible for decision-making on behalf of a large jurisdiction or organization, and we did not include studies related to decision-making for an individual person or patient [15].

We included studies of interventions examining any type of “friendly front end”, “evidence summary”, or “policy brief” or other products derived from systematic reviews or guidelines based on systematic reviews that present evidence in a summarized form to policy-makers and health system managers. Interventions had to include a summary of a systematic review and be actively “pushed” to target users. We included any comparisons including active comparators (e.g., other summary formats) or no intervention.

#### Primary outcomes

1. Use of systematic review derivative product in decision-making (e.g., self-reported use of the evidence in policymaking, decision-making, as well as self-reported access of research, appraisal of research, or commissioning of further research within the decision-making process [16]). We included any type of use including instrumental use of research in decision-making (e.g., direct use of research), as well as conceptual use (e.g., using research to gain an understanding of a problem or intervention), and symbolic use (e.g., using research to confirm a policy/program already implemented) [17]
2. Understanding, knowledge, and/or beliefs (e.g., changes in knowledge scores about the topic included in the summary)

#### Secondary outcomes

- Perceived relevance of systematic review summaries
- Perceived credibility of the summaries
- Perceived usefulness and usability of systematic review summaries
  - o Perceptions and attitudes regarding the specific components of the summaries and their usefulness
- Understandability of summaries
- Desirability of summaries (e.g., layout, selection of images, etc.) [5]

Since some studies may use different terms to describe these outcomes, our team assessed each outcome and categorized them according to the above list.

Two reviewers independently screened titles and abstracts to identify relevant studies meeting the pre-specified inclusion criteria. The full text of each potentially included study was then screened independently by two authors.

### Potential effect modifiers and reasons for heterogeneity

Meta-analysis was not possible, but if it had been, we planned to explore heterogeneity using forest plots and the *I* ^2^ statistic according to guidance of the Cochrane Handbook for Systematic Reviews of Interventions [18]. We were also thus unable to conduct planned meta-regression to assess the role of mediating factors, such as target audience of summary (e.g., focused on specific local context, generic summary), type of decision maker (e.g., federal policy-maker versus hospital administrator), and components of friendly front end (e.g., bulleted list, text, summary of findings table, causal chain).

### Study quality assessment

The methodological quality was assessed using the risk of bias tool from the Cochrane Handbook for randomized trials. If we had identified eligible ITS, CBA, or NRS, we planned to use the Effective Practice and Organization of Care (EPOC) Review Group criteria for ITS and CBA studies [18, 19] and Risk Of Bias In Non-randomized Studies-of Interventions (ROBINS-I) [20, 21].

We used the Grading of Recommendations Assessment, Development, and Evaluation (GRADE) approach to assess the quality of evidence for the outcomes reported in this review [22].

### Data extraction strategy

The data extraction form was pre-tested, and included factors related to the population, intervention, comparison, and outcomes. Data extraction was completed by two authors independently using a structured Excel sheet. Disagreements on extractions were resolved by discussion and with a third member of the research team when necessary. The complete list of data extraction items has been reported elsewhere [13].

### Data synthesis and presentation

Since it was not possible to combine the studies, we have presented the results for each study separately. We planned to conduct sensitivity analyses to assess the effects of incorporating these corrected analyses in our analysis. However, since we did not conduct a meta-analysis, this was not possible. We contacted the corresponding author of studies by email to ask for clarification on missing data and to ask for complete study results for eligible protocols.

## Results

### Review statistics

#### Results of the search

The search strategy yielded 11,733 references (10,113 after removal of duplicates). Figure 1 depicts the results of the search and screening. During the title and abstract screening process, we excluded the 10,059 references for failing to meet one or more of our inclusion criteria. The remaining 50 references were reviewed as full text plus three additional references identified through reference list checking and one additional reference identified through gray literature searching. We excluded 45 studies that did not meet our eligibility criteria (see Additional file 3). We included six completed RCTs (reported in seven articles) in this review [23–28]. The characteristics of the included studies are summarized in Table 1.

**Table 1:** Characteristics of included studies

Study ID	Methods	Participants	Intervention description	Outcomes
Brownson 2011 [23]	RCT	Legislative staff members (e.g., committee staff), state legislators, and executive branch administrators (e.g., division directors, program heads)	4 different policy briefs on mammography screening to reduce breast cancer mortality - Data-focused brief with state-level data - Data-focused brief with local-level data - Story-focused brief with state-level data - Story-focused brief with local-level data Each participant was emailed 1 of the 4 briefs.	Self-reported understandability (using 3 measures assessing whether the information was presented clearly in an attractive way and held the reader’s attention) and credibility (2 measures that assessed whether the information in the brief was believable and accurate)
Carrasco-Labra 2016 [30]	RCT	Health care professionals, guideline developers and researchers that use and/or develop systematic reviews	An alternate summary of findings table was compared against the current format - Alternate format provides options to display the same data in a different way or to provide supplementary data to the current format	Self-reported understanding assessed with 7 multiple choice questions (5 response options). Self-reported accessibility of information assessed with 3 self-reported domains (how easy it is to find critical information, how easy it is to understand the information, whether the information is presented in a useful way for decision-making. Satisfaction measured by asking which about satisfaction with the different formatting elements. Preference assessed using a 7-point Likert scale for the 2 tables
Dobbins 2009 [25]	RCT	Front line staff, managers, directors, coordinators, and others from public health departments in Canada (those directly responsible for making program decisions related to healthy body weight promotion in children)	1st group (control) - Access to health-evidence.ca and received an email about access to this resource 2nd group - Received tailored, targeted messages—7 emails with titles of 7 high-quality SRs related to health body weight promotion in children and links to full text, abstract, and summary, plus access to health-evidence.ca 3rd group - Same intervention as the 2nd group plus access to a full-time knowledge broker who was available to ensuring relevant research, was provided to the decision makers in a way that was useful, helped them to develop skills for evidence-informed decision-making, and translating the evidence	Self-reported global evidence-informed decision-making (participants were asked to report the extent to which research evidence was considered in a recent program planning decision within the previous 12 months) related to healthy body weight promotion and public health policies and programs measured by the sum of actual strategies, policies, and/or interventions for healthy body weight promotion in children being implemented by the department
Masset 2013 [26, 29]	RCT	Individuals who normally read policy briefs related to international development, e.g., employed in academia, NGOs, and international aid organizations, some self-reported influence on policy decisions and therefore considered policymakers	3 versions of a policy brief summarizing the results of a SR - One group received a standard policy brief - 2nd group received a policy brief with director’s commentary - 3rd group received the policy brief with unnamed research fellow’s commentary	Beliefs about the effectiveness of and strength of the evidence for the interventions included in the briefs
Opiyo 2013 [27]	RCT	Panel of healthcare professionals with roles in neonatal and pediatric policy and care in Kenya	3 intervention packages - Pack A contained a systematic review alone - Pack B included systematic reviews with summary of findings tables - Pack C received an evidence summary with a graded entry format	Self-reported understanding of the summary content measured by the proportion of correct responses to clinical questions relevant to the effects of the intervention. Value and accessibility (usefulness and usability) of the evidence was assessed using a 3- or 5-point scale
Vandvik 2012 [28]	RCT	All panelists for the antithrombotic therapy and prevention of thrombosis, American College of Chest Physicians	2 formats of the evidence profile that differed by 4 features - Placement of additional information - Placement of overall quality of evidence - Study event rates - Absolute risk differences Each group received 1 of 4 emails with similar text but different links allowing download of the evidence profile	User preferences for specific formatting options and the overall format of the table were assessed using a 7-point Likert scale Comprehension of key findings was assessed with multiple choice questions Accessibility of the information for quality of evidence and relative and absolute effects was assessed using 3 domains: easy to find, easy to understand, and helpful in making recommendation using a 7-point scale Time needed to comprehend information about quality assessment and key findings was assessed by asking participants to record the time before and after answering questions testing comprehension

The completed studies recruited participants from Canada (*n* = 1), Kenya (*n* = 1), USA (*n* = 1), internationally unspecified countries (46% from high-income countries) (*n* = 1), and countries in Europe, North America, South America, Africa, and Asia (*n* = 1) [23, 25–27, 29, 30]. One study did not report the participants’ country [28]. Additionally, we identified two protocols for eligible studies: one RCT [31] and one CBA [32]. These ongoing studies will be conducted in Canada (*n* = 1) and UK (*n* = 1) [31, 32]. The details of these studies are presented in Table 2.

**Table 2:** Characteristics of ongoing studies

Study ID	Methods	Participants	Intervention description	Outcomes
Wilson 2011 [31]	RCT	Decision makers (programs, services, advocacy) from community-based HIV/AIDS organizations in Canada affiliated with the Canadian AIDS Society and from relevant provincial HIV/AIDS networks	At baseline, all participants will receive the “self-serve” evidence service (includes a listing of relevant systematic reviews, links to PubMed records, and worksheets to help find and use research evidence). During the intervention, one group will receive the “full-serve” version of SHARE (Synthesized HIV/AIDS Research Evidence) which includes access to a database of HIV systematic reviews, emailed updates, access to user-friendly summaries, links to scientific abstracts, peer relevance assessments (indicating how useful the information is), as well as an interface for comments in the records, plus links to the full text, and access to worksheets to help find and use evidence. The control group will continue to receive the self-serve evidence service. During the final 2-month period, both groups will receive the full-serve version of SHARE	The primary outcome measure will be the mean number of logins/month/organization. The secondary outcome will be intention to use research evidence (measured with a survey administered to one key decision maker from each organization)
Wilson 2015 [32]	CBA	Clinical Commissioning Groups: governing body and executive members, clinical leads, and any other individuals deemed as being involved in commissioning decision-making processes	3 arms: (1) consulting plus responsive push of tailored evidence (access to an evidence briefing service provided by the Centre for Reviews and Dissemination (CRD) plus advice and support via phone, email, face-to-face; monthly check in to discuss further evidence needs; issues around use of evidence; alert team to new SRs, and other synthesized evidence relevant to priorities); (2) consulting plus an unsolicited push of non-tailored evidence (access to intervention 1 without tailored evidence briefings and instead just evidence briefings without contextual information); or (3) “standard” service (CRD will disseminate evidence briefings generated in intervention 1 and any other non-tailored briefings produced by CRD over the intervention period)	Primary outcome: change at 12 months from baseline of a CCGs ability to acquire, assess, adapt, and apply research evidence to support decision-making. Secondary outcomes will measure individuals’ intentions to use research evidence in decision-making

### Description of included studies

Details of the different evidence summary formats are reported in Table 3. Briefly, two studies assessed policy briefs [23, 26]; one assessed an “evidence summary” [25]; two assessed different formats of summary of findings tables, which are distinct table formats presenting the main findings of the review (absolute and relative effects for each important outcome) and quality of the evidence [28, 30]; and one compared an SOF table alone to a summary of findings table as part of a “graded entry” evidence summary (a short one-page summary, then a narrative report, followed by access to the complete systematic review) [27]. Two studies assessed evidence summaries which included recommendations for programs or policies [23, 25], while the others did not specify whether recommendations were provided within the summary [26–28].

**Table 3:** Evidence summary formats and results

Study	Type of evidence summary	Format of summary	Method of delivery	Components	Outcomes
Brownson 2011 [23]	Policy brief	Printed leaflet/booklet, PDF version for those who prefer online	Mailed, follow up telephone call, emailed if preferred	Front cover varied according to story- versus data-driven, color printed (included data or story), 3rd and 4th pages are the same across all 4 briefs, data-driven briefs contained 2 statements with percentages related to mammography screening, story-driven had 2 personal stories related to mammography, all briefs had data about uninsured women, women not up to date on mammograms, breast cancer mortality compared to other causes, benefits of mammograms, and recommendations	The briefs were considered understandable and credible (mean ratings ranged from 4.3 to 4.5 on 5.0 Likert scale). Likelihood of using the brief was different by study condition for staff members (*p* = 0.041) and legislators (*p* = 0.018). Staff members found the story-focused brief with state-level data the most useful. Legislators found the data-focused brief with state-level data the most useful
Carrasco-Labra 2016 [30]	Summary of findings table	Table	Emailed link to online survey	The new format of summary of findings table moved the number of participants and studies to the outcomes column, quality of evidence was presented with the main reasons for downgrading, “footnotes” was changed to “explanations”, baseline risk and corresponding risk were expressed as percentages, column presenting absolute risk reduction (risk difference) or mean difference, no comments column, addition of “what happens” column, no description of the GRADE evidence definitions	Participants with the new summary of findings table format had higher proportion of correct answers for almost all questions. The new format was more accessible (easier to understand information about the effects (MD 0.4, SE 0.19); and displayed results in a way that was more helpful for decision-making (MD 0.5 SE 0.18); overall, participants preferred the new format (MD 2.8, SD 1.6)
Dobbins 2009 [25]	Evidence summaries	Text	Targeted, tailored emails	Short summary including key findings and recommendations	The post-intervention change in Global Evidence-Informed Decision-making was 0.74 (95% CI 0.26–1.22) for the group receiving only access to healthevidence.ca; –0.42 (–1.10, 0.26) for the group receiving tailored, targeted emails; and –0.09 (–0.78, 0.60) for the knowledge broker group. The changes in health policies and programs (HPP) after the intervention were –0.28 (–1.20, 0.65) for the group receiving only access to the healthevidence.ca website; 1.67 (0.37, 2.97) for the group receiving tailored, targeted messages; and –0.19 (–1.50, 1.12) for the group with access to a knowledge brokers. The tailored, targeted messages are more effective than the knowledge broker intervention or access to www.health-evidence.ca in organizations with a culture that highly values research
Masset 2013 [26, 29]	Policy brief	Text, colored leaflet	Email	Introduction to the problem, description of methodology, conclusions, and policy implications, 2 versions had expert commentary	Respondents with stronger beliefs about the agricultural interventions at baseline rated the policy brief more favourably The policy brief was less effective in changing respondents’ ratings of the strength of the evidence and effectiveness of the intervention
Opiyo 2013 [27]	Summary of findings table, graded entry summary of evidence	Text, tables	Email	Summary of findings table Graded entry format included a summary and interpretation of main findings and conclusions, a contextually framed narrative report, and summary of findings table	No differences between groups in the odds of correct responses to key clinical questions Both packs B and C improved understanding. Pack C compared to pack A was associated with a significantly higher mean “value and accessibility” score. Pack C compared to pack A was associated with a 1.5 higher odds of judgments about the quality of evidence being clear and accessible. More than half of participants preferred narrative report formats to the full version of the SR (53% versus 25%). A higher respondent percentage (60%) found SRs to be more difficult to read compared to narrative reports, but some (17%) said that SRs were easy to read. About half of the participants (51%) found SRs to be easier to read compared to summary of findings tables (26%)
Vandvik 2012 [28]	Summary of findings table	Table	Email	Tables presented outcomes, number of participants, summary of findings, and quality assessment using GRADE	Participants liked presentation of study event rates over no study event rates, absolute risk differences over absolute risks, and additional information in table cells over footnotes. Panelists presented with time frame information in the tables, and not only in footnotes, were more likely to properly answer questions regarding time frame and those presented with risk differences, and not absolute risks were more likely to rightly interpret confidence intervals for absolute effects. Information was considered easy to find and to comprehend and also helpful in making recommendations regardless of table format

Carrasco-Labra et al. compared a standard format summary of findings table to a new format that presented some of the data in a different way as well as provided supplementary data [30]. All the other included studies tested evidence summary formats using multiple arms. Brownson et al. compared four versions of a policy brief: a state-level, data-focused brief; a local-level, data-focused brief; a story-focused brief with a state-level data; and a story-focused brief with a local-level data [23].

Dobbins et al. had three groups. The first had access to the online database, the second received targeted, tailored messages in addition to access to an online database, and the third group received the same intervention as the second group plus access to a full-time knowledge broker [25].

Masset et al. and the companion paper by Beynon et al. assessed three versions of a policy brief. The first was the standard policy brief, the second was the same policy brief with an additional commentary by a sector expert (the Director of the institution who conducted the review), and the third was the same except the commentary was attributed to an unnamed research fellow [26, 29].

The study by Opiyo et al. compared a systematic review alone to a systematic review with a summary of findings table and a graded entry format that included a short interpretation of the main findings and conclusions (with a summary of findings table), a contextually framed narrative report, and the full systematic review [27].

Finally, the study by Vandvik et al. compared two versions of summary of findings tables with or without four formatting modifications (the placement of additional information, the placement of the overall rating for quality of evidence, the study event rates, and the absolute risk differences) [28].

### Study quality assessment

**Fig. 2:**
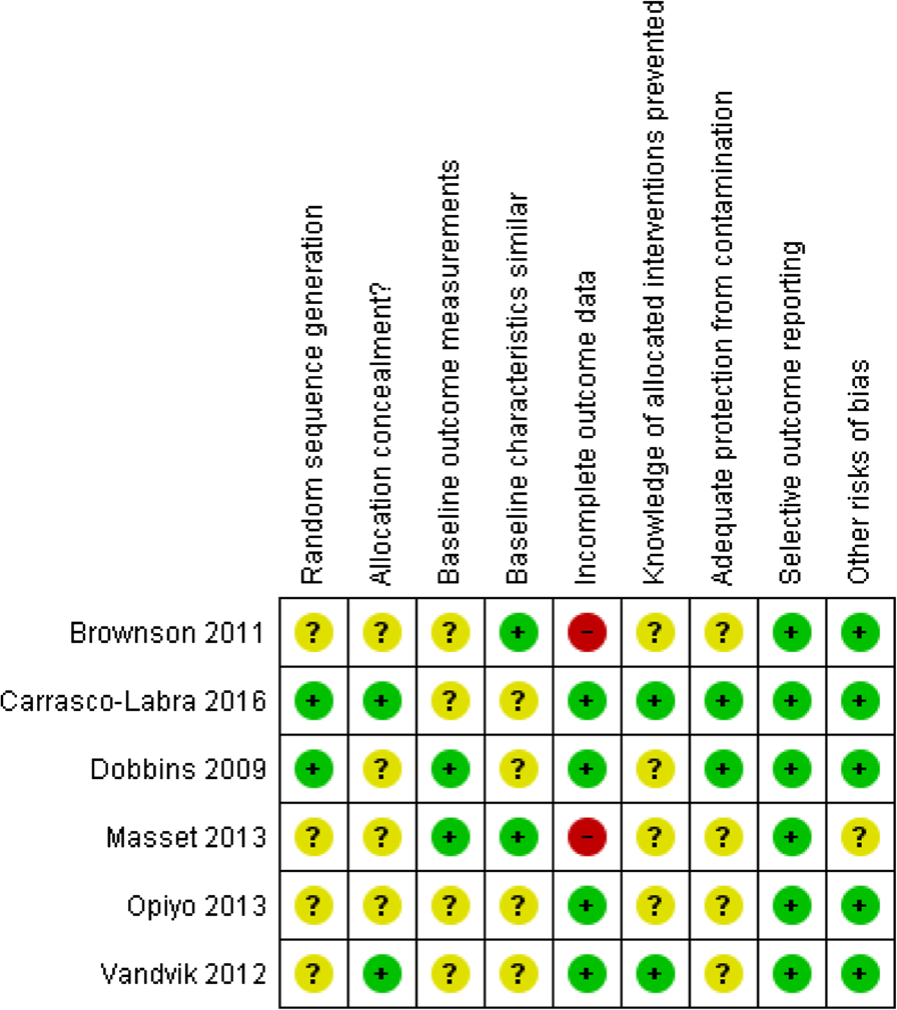
Risk of bias

The summary of the Risk of Bias assessments is presented in Fig. 2. Two studies were assessed as low risk of bias for random sequence generation [25, 30], and the others were assessed as unclear [23, 26–28]. For allocation concealment, four studies were assessed as unclear [23, 25–27, 29] and two studies assessed as low risk of bias [28, 30]. Baseline outcome measurements were similar and therefore low risk of bias in two studies [25, 26, 29] and unclear in four [23, 27, 28, 30]. Baseline characteristics were also similar in two studies [23, 26, 29] and unclear in the others [25, 27, 28, 30]. Incomplete outcome data was assessed as low risk of bias for four studies [25, 27, 28, 30] but high for two studies [23, 26, 29]. These two studies had very high rates of attrition; Brownson et al. had an overall response rate of 35%, and the Masset study had 50% attrition between the baseline and first follow-up [23]. Prevention of knowledge of allocated interventions was assessed as unclear for four of the studies [23, 25–27, 29]. One study reported that panelists, data collection, and data analysis were blinded [28], and one reported that allocation was done in real time when the survey was completed, and these were therefore assessed as low risk of bias [30]. Adequate protection from contamination was assessed as unclear for four studies. The Dobbins study included public health departments from across Canada, and therefore, little risk of contamination was expected [25], and Carrasco-Labra et al. reported that allocation was done in real time when completing the survey leaving little risk of contamination [30]. All studies were assessed as low risk of bias for selective outcome reporting.

Most outcomes were assessed as moderate certainty of evidence using GRADE [22]. The reasons for downgrading the evidence were due to unclear risk of bias or small sample sizes. Perceived desirability of the summaries was assessed as high certainty of evidence. The assessments are included in Table 4.

**Table 4:** Summary of findings table

Evidence summaries to increase policymakers’ use of systematic review evidence			
Patient or population: policymakers and health system managers Settings: Intervention: evidence summaries based on systematic review Comparison: any comparison			
Outcomes	Impact	No. of participants (studies)	Quality of the evidence (GRADE)
Use of systematic review evidence in decision-making	Little to no difference in effect on evidence-informed decision-making when compared to access to a knowledge broker or online registry of research [25] Little to no difference in effect on self-reported likelihood of using data-driven versus story-driven policy briefs (with state-level or local-level data) [23]	399 (2)	⊕⊕⊕⊝ Moderate^a^
Understanding, knowledge and/or beliefs	One study found little to no effect on understanding of information when provided in different summary of findings table formats [28] while the other found that those provided with a new version of the summary of findings table had consistently higher proportions of correct answers assessing understanding of key findings provided in the table [30] Little to no effect in understanding of information for a graded entry format compared to an summary of findings table or systematic review alone [27] Little to no effect on changing participants’ beliefs about the strength of the evidence for those who already had beliefs but increased the number of participants who had beliefs about the strength of the evidence [26, 29]	676 (4)	⊕⊕⊕⊝ Moderate^a^
Perceived credibility of the summaries	Little to no difference in perceived credibility for different versions of the policy brief (data-driven versus story-driven, local- versus state-level data) [23]	291 (1)	⊕⊕⊕⊝ Moderate^a^
Perceived usefulness and usability of systematic review summaries	The graded entry format was rated higher than the systematic review alone, and there was little to no difference between the ratings for the summary of findings table and the systematic review alone [27] Different summary of findings table formats had little to no effect in one study [28], but a new summary of findings format was found to be more accessible than the standard summary of findings in another [30]	443 (3)	⊕⊕⊕⊝ Moderate^a^
Perceived understandability of the summaries	All formats of the policy brief were reported as easy to understand [23] Graded entry formats were easier to understand the summary of findings tables or systematic reviews alone [27]	356 (2)	⊕⊕⊕⊝ Moderate^a^
Perceived desirability of the summaries	Alternate versions of the summary of findings were preferred [28, 30]	378 (2)	⊕⊕⊕⊕ High

*GRADE* working group grades of evidence, *High quality* further research is very unlikely to change our confidence in the estimate of effect, *Moderate quality* further research is likely to have an important impact on our confidence in the estimate of effect and may change the estimate, *Low quality* further research is very likely to have an important impact on our confidence in the estimate of effect and is likely to change the estimate, *Very low quality* we are very uncertain about the estimate

^a^Unclear ROB

### Evidence of effectiveness

We generated a summary of findings table for this review (Table 4). This is a narrative summary of all studies assessing a particular outcome domain, pooled across different policy brief formats.

#### Primary outcomes

##### Use of summaries in decision-making

Two studies assessed self-reported use of summaries in decision-making. First, Dobbins et al. assessed instrumental use; the change in global evidence-informed decision-making (EIDM) (defined as the extent to which research evidence was considered in a recent decision) after 18 months. The authors found that the intervention had no significant effect on EIDM. This study also reported on evidence-based public health policies and programs as a measure of the actual number of strategies, policies, and interventions for health body weight promotion among children that were implemented by the health department. For this outcome, the group that received the targeted, tailored messages had a significant increase in evidence-based public health policies and programs.

The study by Brownson et al. asked policymakers how likely they would be to use the evidence summary in decision-making classified as conceptual use of research. On a five-point Likert scale (where 1 is strongly disagree and 5 is strongly agree), there was little to no difference based on the type of policy brief (data-driven versus story-driven) (range 3.3 to 3.4). However, there were differences in self-reported likelihood of using the policy brief depending on the type of policymaker. Staff members reported being the most likely to use the story-focused brief with state-level data (mean rating of 3.4, 95% confidence interval (CI) 3.0 to 3.9) and the least likely to use the data-focused brief with state-level data (2.5, 95% CI 2.0 to 3.0). Legislators reported being the most likely to use the data-focused brief with state-level data (4.1, 95% CI 3.6 to 4.6) and the least likely to use the story-focused brief with state-level data (3.1, 95% CI 2.6 to 3.6) [23].

##### Understanding, knowledge, and/or beliefs

Carrasco-Labra et al. found that respondents receiving the new summary of findings format had a higher proportion of correct answers for almost all questions. These included the ability to interpret footnotes (risk difference (RD) 7%, *p* = 0.18), ability to determine risk difference (RD 63%, *p* = <0.001), understanding of quality of evidence and treatment effect (RD 62%, *p* = <0.001), understanding of the quality of evidence (RD 7%, *p* = 0.06), and ability to quantify risk (RD 6%, *p* = 0.06) [30]. However, for one question, the ability to relate the number of participants and studies to outcomes, the group receiving the standard summary of findings scored slightly higher (RD −3%, *p* = 1.0).

The Masset study examined changes in beliefs about the effectiveness of the intervention as well as the strength of the evidence included in the policy briefs. The authors found that the policy brief increased the number of participants who had an opinion about the strength of the evidence (e.g., those who did not have an opinion at baseline formed an opinion based on the policy brief) but was less effective in changing participants’ ratings of the strength of the evidence or the effectiveness of the intervention [26]. The policy brief did not change the opinions of those who had an opinion at baseline about the evidence and effectiveness.

The Opiyo study found little to no difference between the interventions for the odds of correct responses and questions about the intervention (adjusted odds ratio (OR) for summary of findings table compared to systematic review alone was 0.59, 95% CI 0.32 to 1.07, and for graded entry format compared to systematic review alone OR 0.66, 95% CI 0.36 to 1.21); however, both of these indicated that the odds of correct responses were higher for the groups who received an evidence summary or summary of findings [27]. However, when comparing groups of participants, both the summary of findings tables and the graded entry formats slightly improved understanding for policymakers (summary of findings table compared to systematic review alone adjusted OR 1.5, 95% CI 0.15 to 15.15 and for graded entry format compared to systematic review alone, 1.5 (0.64 to 3.54)) [27].

Finally, Vandvik et al. reported that there was little to no difference in participants’ understanding of information in the different table formats for most items (range 80 to 97% for table A compared to 69 to 92% for table B, *p* values from 0.26 to 0.86) However, those with table A had higher scores for two items: time period for risk estimates (58% compared to 11%, *p* < 0.0001) and the range in which the effect may lie (95% versus 54%, *p* < 0.0001) [28].

#### Secondary outcomes

##### Credibility of the summaries

Brownson et al. reported little to no differences in credibility between the different intervention formats. The mean scores for perceived credibility ranged from 4.4 to 4.5 on a five-point Likert scale [23]. For different policymaker groups, there were also little to no differences with mean scores ranging from 4.2 to 4.5 for staff members, 4.3 to 4.7 for legislators, and 4.3 to 4.6 for executives [23].

The Masset study assessed how convincing the policy brief was, how robust the methodology was, and how strong the evidence was. Participants who had stronger beliefs about the evidence at baseline rated the policy brief more favorably [26].

##### Perceived usefulness and usability of the summaries

The Carrasco-Labra study reported that the new summary of findings format was more accessible than the standard format [30]. This was assessed by asking respondents about their ease of finding the information about the effects (MD 0.4, SE 0.19, *p* = 0.04) and ease of understanding the information (MD 0.5, SE 0.2, *p* = 0.011). The respondents also reported that the new format displayed results in a way that was more helpful for decision-making (MD 0.5, SE 0.18, *p* = 0.011).

Opiyo et al. measured this outcome by assessing the “value and accessibility” of each intervention. The graded entry format received a higher mean score than the systematic review alone (adjusted mean difference (MD) 0.52 (95% CI 0.06 to 0.99). There was little to no difference in effect when comparing the summary of findings table and the systematic review alone (MD −0.11, 95% CI −0.71 to 0.48) [27].

Vandvik et al. reported that accessibility of information for quality of evidence as well as absolute and relative effects was rated similarly with no significant differences between groups [28]. Only pooled results were presented.

##### Perceived understandability of the summaries

All the groups in the Brownson et al. study reported that the summaries were easy to understand [23]. The mean ratings ranged from 4.3 to 4.4 on a five-point Likert scale. For the different policymaker groups, there was little to no difference with mean scores ranging from 4.3 to 4.5 for staff members and legislators and 4.1 to 4.4 for executives [23].

The study by Opiyo et al. reported that 60% (95% CI 48 to 73%) of the participants found systematic reviews to be more difficult to read than the narrative reports included in the graded entry formats. Fifty-one percent (95% CI 38 to 63%) compared to 26% (95% CI 15 to 37%) found the systematic reviews to be easier to read than the summary of findings tables, while 53% (95% CI 41 to 65%) compared to 25% (95% CI 14 to 36%) preferred the narrative report format (graded entry) to the full systematic review [27].

##### Perceived desirability of the summaries

The two studies of different summary of findings formats assessed this outcome. One study found that participants preferred the presentation of study event rates versus not having them (median 1, interquartile range (IQR) 1, on 1–7 scale, where 1 was strong preference for and 7 was strong preference against), absolute risk differences versus presentation of absolute risks (median 2, IQR 3), and having the additional information embedded in table versus having it as footnotes (median 1, IQR 2). No significant differences found for the placement of the column for overall quality of evidence (either as the final column or before the effect size) or the overall table format (differing by column headings and order of columns) [28].

The other study found that overall, respondents preferred the new summary of findings format (MD 2.8, SD 1.6) [30].

None of the included studies reported on policymakers’ perceived relevance of the summaries.

### Effect modifiers

The organizational research culture was found to influence the effect of the intervention on evidence-based public health policies and programs in one study which found that tailored, targeted messages were more effective than access to a database alone (healthevidence.ca) or access to a knowledge broker when the organization valued research evidence in decision-making [25].

The Carrasco-Labra study found that the number of years of experience of the respondents modified the effect on understanding by more than 10% (adjusted OR 1.83; 95% CI 0.91 to 3.67) for the questions about the ability to determine a risk difference. For the question assessing whether respondents understand the quality of evidence and treatment effect combined, the authors found that years of experience, familiarity with GRADE, and level of training modified the effect by more than 10% (adjusted OR 0.72; 95% CI 0.20 to 2.56).

## Discussion

This review has summarized the evidence on the use of systematic review summaries in policymaking, policymakers’ understanding of systematic review evidence, and different components and design features. Overall, the results suggest that evidence summaries are likely easier to understand than complete systematic reviews. However, their ability to increase the use of systematic review evidence in policymaking is unclear. Six studies were included in this review. For our primary outcome, the use of systematic review evidence in decision-making, we found moderate certainty of evidence. One study found that targeted, tailored messages increased the number of evidence-based public health policies and programs; however, for the two studies that assessed effect on decision-making or likelihood of using the summary in decision-making, there was little to no difference between the intervention groups [23, 25]. For the secondary outcome, understanding, knowledge, and beliefs, there was little to no difference in effect and moderate certainty of evidence in three of the four studies assessing this outcome [26–28] although there was a slight increase in understanding for summary of findings tables and graded entry formats compared to systematic reviews alone. The fourth study found that those provided with an alternate version of the summary of findings had greater understanding [30]. For perceived desirability of summaries, we found high certainty of evidence. In summary of findings tables, one study found that the alternate version was preferred, [30] and the other study found that certain formatting elements were preferred such as study event rates, absolute risk differences, and additional information provided in the table and not in footnotes [28]. One study found the alternate format to be more accessible than the standard format [30]; however, the other study assessing formatting changes found little to no difference in effect for perceived usefulness [28]. For perceived usefulness and usability of the summaries, we found low certainty of evidence. The graded entry summary was rated higher than a systematic review alone for usability [27]. Summaries were perceived as easier to understand than systematic reviews (moderate certainty of evidence) [23, 27]. There was little to no difference in effect for different versions of the policy brief (data-driven versus story-driven, local- versus state-level data; moderate certainty of evidence) for perceived credibility of the summaries [23].

Our primary outcome, the policymakers’ use of systematic review evidence in decision-making, is challenging to measure. Other studies have noted the inherent challenges in measuring this outcome since many factors contribute to decision-making, and it is often difficult for an individual to identify which factors had a role in their final decision [25, 33]. Instead of determining the actual use of research in decision-making, studies assessed self-reported use of research or other outcomes, such as perceived credibility or relevance, since these may affect the likelihood of research use in decision-making.

Our review is limited by the indexing of studies in this area. To address this issue, we conducted a broad search using search strategies adapted from similar systematic review. Our search identified over 10,000 references, but we had a low yield of included studies. The methods used in the included studies were poorly reported. For example, only two studies adequately reported on random sequence generation or allocation concealment, which means that most studies have unclear risk of bias.

More research is needed to determine whether evidence summaries can increase the use of systematic reviews by policymakers and health system managers. We identified two protocols for ongoing studies which are promising as the results of these studies will enhance the available evidence about the effectiveness of evidence summaries [31, 32]. We also identified other relevant studies assessing the effectiveness of systematic review derivatives that did not use an eligible study design (e.g., used interviews or other methods without a control group) [5, 21]. One of these studies was intended to be a RCT and process evaluation but was not eligible for our review because poor recruitment (only 15% of the planned sample) resulted in the termination of the trial [21]. This demonstrates the difficulty with recruiting these types of participants. Recruitment for the process evaluation remained low, and the authors noted that those included are likely those already more interested in using systematic review derivatives [21]. The authors noted that for future RCTs, recruitment may be more successful achieved from randomizing divisions versus individuals since the nature of policymaking is quite complex and often not completed at the individual level. Additionally, we identified other studies that were not focused on policymakers but rather, clinicians [34, 35] or the public [36]. These studies demonstrated that evidence summaries can improve understanding of research evidence within these populations; however, use of evidence in decision-making was not assessed.

It is important to note that only two of the included studies compared the evidence summary to a full systematic review or access to a database of systematic reviews. The others compared different versions of evidence summaries and, in general, found little to no differences in the effects. Had these studies included systematic reviews as a control group, the results may be different. Additional research on the use of evidence summaries derived from systematic reviews is needed.

A previously conducted systematic review identified poor access to high-quality and relevant research as a barrier to the use of research evidence by policymakers [12]. Evidence summaries can address this barrier by increasing access to systematic review evidence provided that policymakers are aware that these products are available. Our review has not identified the best way to disseminate these products although one study found that targeted, tailored messages improved research use by the policymakers [25]. Future developers of systematic review products should collaborate with the policymakers to ensure that their summaries are relevant to those making decisions in practice [12]. Future studies should include an assessment of delivery strategies since the effectiveness of the systematic review derivative product in practice will be impacted by the policymakers’ knowledge of and access to the summaries themselves. Our included studies suggest that evidence summaries have a small effect on improving knowledge and understanding and should be created. However, we have very little evidence to inform the design of evidence summaries since we only found a handful of different formats (none the same), and there was little to no difference between formats when compared directly.

The interventions assessed in the studies included in our review are quite diverse with a variety of outcome measures. We included a broad range of interventions to provide an overview of the evidence on systematic review derivative products. These products have important differences in design and source material. For example, a policy brief includes evidence from one or more systematic reviews and includes information from additional sources [4, 7], whereas a summary of findings table reports results for a single systematic review. We chose to include all systematic review derivative products since there are limited studies on a single product type. We recognize that this creates a challenge for interpreting the results since the interventions were quite different. Therefore, we have provided a narrative summary of each study and presented an overview of the available evidence.

## Additional files

Additional file 1: MEDLINE Search Strategy. (DOCX 17 kb)
Additional file 2: Grey Literature Searches. (DOCX 15 kb)
Additional file 3: Excluded Studies. (DOCX 59 kb)

## Abbreviations

3ie: International Initiative for Impact Evaluation
CBA: Controlled before-after studies
CIHR: Canadian Institutes of Health Research
COMMVAC: Communicate to vaccinate
DfiD: Department for International Development
GRADE: Grading of Recommendations Assessment, Development, and Evaluation
ICC: Intracluster correlation coefficient
ITS: Interrupted time series
NGO: Non-governmental organization
NRCT: Non-randomized controlled trial
RCT: Randomized controlled trial
WHO: World Health Organization
