# Supplementary material for: The effectiveness of evidence summaries on health policymakers and health system managers use of evidence from systematic reviews: a systematic review
Source: Implement Sci. 2016 Dec 9;11:162. doi: 10.1186/s13012-016-0530-3 (PMC5148903; doi:10.1186/s13012-016-0530-3)
Supplement: Additional file 1: — MEDLINE Search Strategy. (DOCX 17 kb) [file 13012_2016_530_MOESM1_ESM.docx]

**Additional File 1**

**MEDLINE search strategy**

1. ((systematic review$ or methodolog$ review$ or quantitativ$ review$ or qualitativ$ review$ or overview$ or synthes$ or metasynthes$ or megasynthes$) adj5 (decisionmak$ or decision-mak$ or policy-mak$ or policymak$ or policy decision$ or health$ polic$ or health$ manag$ or action$ or commission* or purchas* or procur* or budget hold* or budgethold* or service provi*or practice or application or implement$ or utili?ation or utili?ing or utili$ or disseminat$ or summar$ or hospital* decision* or treatment plan* or patient care or patientcare or healthcare or health care or clinical decision* or pathway* or algorithm*)).ti,ab.

2. (((systematic adj2 (review* or overview* or synthesis or literature review* or evidence review*)) ormethodolog* review* or quantitativ* review* or qualitative review* or overview or synthes* or metasynthes* or megasynthes*) adj5 (policy or policies or decision*)).ti.

3. ((gap or gaps) adj7 ((knowledge or research or evidence or trial or result) adj2 practice)).ti,ab.

4. 1 or 2 or 3

5. randomized controlled trial.pt.

6. controlled clinical trial.pt.

7. randomized.ab.

8. placebo.ab.

9. clinical trials as topic/

10. randomly.ab.

11. trial.ti.

12. intervention*.ti.

13. or/5-12

14. State Medicine/

15. exp Purchasing, Hospital/

16. Contracts/

17. exp Contract Services/

18. exp Organizational Innovation/

19. Insurance, Health/ or exp Managed Care Programs/ or Medicare/

20. (commissioning or commissioner$).ti,ab.

21. (purchasing or purchaser$).ti,ab.

22. (procurement or procurer$).ti,ab.

23. (budget-holder$ or budgetholder$).ti,ab.

24. (service adj2 (development or developer$ or provision or provider$)).ti,ab.

25. ((investment or budget or purchas$ or service) adj3 priorit$).ti,ab.

26. priorit$ setting.ti,ab.

27. decision-maker$.ti,ab.

28. (contract$ adj3 (management or services or tender$)).ti,ab.

29. Decision Making, Organizational

30. exp Policy Making/

31. exp Health Planning/

32. or/14-31

33. exp Evidence-Based Practice/

34. Translational Research/

35. exp 'Diffusion of Innovation'/

36. ((research or knowledge or innovation$ or evidence) adj5 (diffus$ or disseminat$ or implement$ or adoption or exchang$ or application or mobilis$ or mobiliz$ or synthes$ or transfer$ or translat$ or incorporat$ or uptak$ or utilis$ or utiliz$ or transmission or integrat$ or democratis$ or democratiz$ or shar$ or broke$)).ti,ab.

37. ('research into practice' or 'knowledge into practice' or 'knowledge into action' or 'research into action' or 'research findings into action' or 'evidence into action' or 'evidence into practice').ti,ab.

38. (KT adj5 (diffus$ or disseminat$ or implement$ or adoption or exchang$ or application or mobilis$ or mobiliz$ or synthes$ or transfer$ or translat$ or incorporat$ or uptak$ or utilis$ or utiliz$ or transmission or integrat$ or democratis$ or democratiz$ or shar$ or broke$)).ti,ab.

39. ((evidence base$ or evidence inform$) adj5 (decision$ or plan$ or policy or policies or practice or action$)).ti,ab.

40. ((research or knowledge or innovation$ or evidence) adj5 (change$ or changing or improv$ or promot$ or influenc$ or impact$ or disinvest$ or discontinu$ or reject$ or abandon$ or ceas$ or restrict$ or disincentiv$ or stop$)).ti,ab.

41. ((research utiliz$ or research utilis$ or evidence or knowledge or innovation$) adj5 (decision-mak$ or decisionmak$ or policy-mak$ or policymak$ or health$ manag$ or health$ polic$ or action$ or practice or policy decision$)).ti,ab.

42. (('use' or using or usage or useful or utiliz$ or utilis$) adj5 (evidence or research)).ti,ab.

43. Information Dissemination/

44. (disseminat$ adj5 (findings or results)).ti,ab.

45. 'Health Knowledge, Attitudes, Practice'/

46. Attitude of Health Personnel/

47. Clinical Competence/

48. or/34-47

49. ((research or knowledge or innovation$ or evidence or information or policy) adj5 (brief$ or summar$ or synops$ or overview$ or bulletin$ or synthes$ or map or mapping or maps or framing$ or product$ or package$ or alert$ or commentar$ or strateg$ or algorithm$)).ti,ab.

50. (push activit* or pull activit*).ti,ab.

51. (collaborat$ or 'cross-profession$' or intraprofession$ or intra-profession$ or interprofession$ or inter-profession$ or inter-disciplin$ or multi-disciplin$ or multi disciplin$ or multiprofession$ or outsourc$ or subcontract$).ti,ab.

52. 'linkage and exchange'.ti,ab.

53. or/48-51

53. 4 and 13 and (32 or 48 or 53)
