# Supplementary material for: The effectiveness of evidence summaries on health policymakers and health system managers use of evidence from systematic reviews: a systematic review
Source: Implement Sci. 2016 Dec 9;11:162. doi: 10.1186/s13012-016-0530-3 (PMC5148903; doi:10.1186/s13012-016-0530-3)
Supplement: Additional file 2: — Grey Literature Searches. (DOCX 15 kb) [file 13012_2016_530_MOESM2_ESM.docx]

**Additional File 2**

**Grey Literature Sources**

3ie Policy Briefs (<http://www.3ieimpact.org/en/evidence/policy-briefs/>)

Canadian Agency for Drugs and Technology in Health (<https://www.cadth.ca/>)

Capacity Plus (<http://www.capacityplus.org/>)

CDC Community Guide (<http://www.thecommunityguide.org/index.html>)

Communicate to Vaccinate (<http://www.commvac.com/>)

Consortium for Research on Equitable Health Systems (<http://www.crehs.lshtm.ac.uk/>)

Developing and Evaluating Communication Strategies to Support Informed Decisions and Practice Based on Evidence (<http://www.decide-collaboration.eu/>)

Epistemonikos (<http://www.epistemonikos.org/>)

Evidence Aid (<http://www.evidenceaid.org/>)

EVIPNet/SURE (<http://global.evipnet.org/en>) and (<http://www.who.int/evidence/sure/policybriefs/en/>)

Global HIV/AIDS Initiatives Network (GHIN) (<http://www.aidsmap.com/>)

Health Action International (<http://haiweb.org/>)

Health Systems Evidence (<https://www.healthsystemsevidence.org/>)

Human Sciences Research Council (<http://www.hsrc.ac.za/en>)

IntraHealth International/Capacity Project (<http://www.intrahealth.org/page/capacityplus>)

McMaster Health Forum Evidence briefs (<https://www.mcmasterhealthforum.org/>)

Partnership for maternal, newborn, and child health (WHO) (<http://www.who.int/pmnch/en/>)

PDQ evidence (<http://www.pdq-evidence.org/>)

Rx for change (<http://rxforchange.ucsf.edu/>)

Social, Technological and Environmental Pathways to Sustainability Centre (<http://steps-centre.org/#&panel1-1>)

SUPPORT Summaries (<http://supportsummaries.org/>)

WHO - Department of Health Systems Financing (<http://www.who.int/healthsystems/topics/financing/en/>)

WHO - Department of human resources for health (<http://www.who.int/hrh/about/en/> OR <http://www.who.int/hrh/en/>)

World Bank - Reaching the poor (<https://openknowledge.worldbank.org/handle/10986/7393>)
