# Supplementary material for: The effectiveness of evidence summaries on health policymakers and health system managers use of evidence from systematic reviews: a systematic review
Source: Implement Sci. 2016 Dec 9;11:162. doi: 10.1186/s13012-016-0530-3 (PMC5148903; doi:10.1186/s13012-016-0530-3)
Supplement: Additional file 3: — Excluded Studies. (DOCX 59 kb) [file 13012_2016_530_MOESM3_ESM.docx]

**Additional file 3: Characteristics of Excluded Studies**

| **Ref ID** | **Reason for Exclusion** |
| --- | --- |
| Alper 2005[[1](#_ENREF_1)] | Did not include policymakers |
| Bartels 2011[[2](#_ENREF_2)] | Did not include policymakers |
| Bero 1997[[3](#_ENREF_3)] | The intervention did not assess summaries/derivatives of systematic reviews |
| Carrasco-Labra 2015[[4](#_ENREF_4)] | Protocol for completed study included in review |
| Caruana 2008[[5](#_ENREF_5)] | Not an eligible study design, did not include policymakers |
| Chambers 2011[[6](#_ENREF_6)] | Not an eligible study design |
| Chambers 2012[[7](#_ENREF_7)] | Not an eligible study design – no control group |
| Coulter 2006[[8](#_ENREF_8)] | Not an eligible study design |
| Dobbins 2001[[9](#_ENREF_9)] | Not an eligible study design |
| Dobbins 2004[[10](#_ENREF_10)] | Intervention included complete systematic review not a derivative product. |
| Dobbins 2007[[11](#_ENREF_11)] | Not an eligible study design |
| Fahey 1995[[12](#_ENREF_12)] | Not an eligible study design |
| Kelechi 2010[[13](#_ENREF_13)] | Not an eligible study design |
| Kendall 2013[[14](#_ENREF_14)] | Not an eligible study design |
| Kirkpatrick 1995[[15](#_ENREF_15)] | Did not include policymakers |
| Lavis 2005[[16](#_ENREF_16)] | Not an eligible study design |
| Lavis 2011[[17](#_ENREF_17)] | Completed study identified but RCT was not conducted because of limited enrollment. The authors instead conducted interviews so this was not an eligible study design. |
| Lorenc 2014[[18](#_ENREF_18)] | Not an eligible study design |
| MacFarlane 2011[[19](#_ENREF_19)] | Not an eligible study design |
| MacGregor 2014[[20](#_ENREF_20)] | Not an eligible study design |
| Madhavan 2012[[21](#_ENREF_21)] | Not an eligible study design |
| Mallory 2010[[22](#_ENREF_22)] | Not an eligible study design |
| Malterud 2016[[23](#_ENREF_23)] | Not an eligible study design |
| Maluka 2014[[24](#_ENREF_24)] | Not an eligible study design |
| Mitchell 2011[[25](#_ENREF_25)] | Not an eligible study design |
| Moat 2013[[26](#_ENREF_26)] | Not an eligible study design |
| Mossialos 2013[[27](#_ENREF_27)] | Not an eligible study design |
| Munn 2015[[28](#_ENREF_28)] | Not an eligible study design |
| Murthy 2012[[29](#_ENREF_29)] | Not an eligible study design- systematic review |
| Nannini 2010[[30](#_ENREF_30)] | Not an eligible study design |
| Noor 2009[[31](#_ENREF_31)] | Not an eligible study design |
| Nutley 2014[[32](#_ENREF_32)] | Intervention assessed did not include systematic review derivatives |
| Oermann 2009[[33](#_ENREF_33)] | Did not include policy makers |
| Oliver 2014[[34](#_ENREF_34)] | Not an eligible study design- systematic review |
| Perrier 2011a[[35](#_ENREF_35)] | Not an eligible study design- systematic review |
| Perrier 2011b[[36](#_ENREF_36)] | Not an eligible study design- systematic review |
| Perrier 2015[[37](#_ENREF_37)] | Did not include policy makers |
| Rosenbaum 2011[[38](#_ENREF_38)] | Not an eligible study design |
| Santesso 2015[[39](#_ENREF_39)] | Did not include policy makers (patients and the public) |
| Sullivan 2014[[40](#_ENREF_40)] | Not an eligible study design |
| Taylor-Robinson 2008[[41](#_ENREF_41)] | Not an eligible study design |
| Thomson 2013a[[42](#_ENREF_42)] | Not an eligible study design |
| Thomson 2013b[[43](#_ENREF_43)] | Not an eligible study design |
| Tricco 2016[[44](#_ENREF_44)] | Scoping review not an eligible study design |
| Wallace 2014 [[45](#_ENREF_45)] | Systematic review not an eligible study design |
| Wilson 2013[[46](#_ENREF_46)] | Not an eligible study design |
| Yavchitz 2014[[47](#_ENREF_47)] | Did not include policy makers |

**References**

1. Alper BS, White DS, Ge B: **Physicians answer more clinical questions and change clinical decisions more often with synthesized evidence: a randomized trial in primary care.** *Ann Fam Med* 2005, **3:**507-513.

2. Bartels S, Unutzer J, Snowden M, Bruce ML: **How can we get research findings into practice? Results from three national programs on implmenting evidence-based practices for geriatric depression.** *Am J Geriatr Psychiatry* 2011, **1**.

3. Bero LA, Jadad AR: **How consumers and policymakers can use systematic reviews for decision making.** *Ann Intern Med* 1997, **127:**37-42.

4. Carrasco-Labra A, Brignardello-Petersen R, Santesso N, Neumann I, Mustafa RA, Mbuagbaw L, Ikobaltzeta IE, De Stio C, McCullagh LJ, Alonso-Coello P, et al: **Comparison between the standard and a new alternative format of the Summary-of-Findings tables in Cochrane review users: study protocol for a randomized controlled trial.** *Trials* 2015, **16**.

5. Caruana E: **Evidence for Nursing Practice Updates from the Joanna Briggs Institute. Summaries of Systematic Reviews on nursing-related issues from the Cochrane Library, Joanna Briggs Institute and other evidence review organizations.** *J Adv Nurs* 2008, **62:**271-272.

6. Chambers D, Wilson PM, Thompson CA, Hanbury A, Farley K, Light K: **Maximizing the impact of systematic reviews in health care decision making: a systematic scoping review of knowledge-translation resources.** *Milbank Q* 2011, **89:**131-156.

7. Chambers D, Grant R, Warren E, Pearson SA, Wilson P: **Use of evidence from systematic reviews to inform commissioning decisions: A case study.** *Evidence & Policy* 2012, **8:**141-148.

8. Coulter ID: **Evidence summaries and synthesis: necessary but insufficient approach for determining clinical practice of integrated medicine?** *Integr Cancer Ther* 2006, **5:**282-286.

9. Dobbins M, Cockerill R, Barnsley J: **Factors affecting the utilization of systematic reviews. A study of public health decision makers.** *Int J Technol Assess Health Care* 2001, **17:**203-214.

10. Dobbins M, Thomas H, O'Brien MA, Duggan M: **Use of systematic reviews in the development of new provincial public health policies in Ontario.** *Int J Technol Assess Health Care* 2004, **20:**399-404.

11. Dobbins M, Jack S, Thomas H, Kothari A: **Public health decision-makers' informational needs and preferences for receiving research evidence.** *Worldviews Evid Based Nurs* 2007, **4:**156-163.

12. Fahey T, Griffiths S, Peters TJ: **Evidence based purchasing: understanding results of clinical trials and systematic reviews.** *BMJ* 1995, **311:**1056-1059; discussion 1059-1060.

13. Kelechi TJ, Naccarato MK: **Knowledge translation: summarizing and synthesizing the evidence for WOC best practices.** *J Wound Ostomy Continence Nurs* 2010, **37:**132-136.

14. Kendall S: **Summaries of evidence: an adjunct to knowledge translation?** *Prim Health Care Res Dev* 2013, **14:**107-108.

15. Kirkpatrick S: **Using information networks as a decision-making tool.** *J Healthc Resour Manag* 1995, **13:**21-24.

16. Lavis J, Davies H, Oxman A, Denis JL, Golden-Biddle K, Ferlie E: **Towards systematic reviews that inform health care management and policy-making.** *J Health Serv Res Policy* 2005, **10 Suppl 1:**35-48.

17. Lavis JN, Wilson MG, Grimshaw JM, Haynes RB, Hanna SE, Raina P, Gruen RL, Ouimet M: **Effects of an evidence service on health-system policy makers’ use of research evidence: A protocol for a randomised controlled trial.** *Implement Sci* 2011, **6**.

18. Lorenc T, Tyner EF, Petticrew M, Duffy S, Martineau FP, Phillips G, Lock K: **Cultures of evidence across policy sectors: systematic review of qualitative evidence.** *Eur J Public Health* 2014, **24:**1041-1047.

19. MacFarlane A, Clerkin P, Murray E, Heaney DJ, Wakeling M, Pesola UM, Waterworth EL, Larsen F, Makiniemi M, Winblad I: **The e-Health Implementation Toolkit: qualitative evaluation across four European countries.** *Implement Sci* 2011, **6:**122.

20. MacGregor JC, Wathen N, Kothari A, Hundal PK, Naimi A: **Strategies to promote uptake and use of intimate partner violence and child maltreatment knowledge: an integrative review.** *BMC Public Health* 2014, **14:**862.

21. Madhavan R, Mahoney JT: **Evidence-Based Management in "Macro" Areas: The Case of Strategic Management.** St. Louis: Federal Reserve Bank of St Louis; 2011.

22. Mallory GA: **Professional nursing societies and evidence-based practice: strategies to cross the quality chasm.** *Nurs Outlook* 2010, **58:**279-286.

23. Malterud K, Bjorkman M: **The Invisible Work of Closeting: A Qualitative Study About Strategies Used by Lesbian and Gay Persons to Conceal Their Sexual Orientation.** *J Homosex* 2016**:**1-16.

24. Maluka S, Kamuzora P, Ndawi B, Hurtig AK: **Involving decision-makers in the research process: Challenges of implementing the accountability for reasonableness approach to priority setting at the district level in Tanzania.** *Glob Public Health* 2014, **9:**760-772.

25. Mitchell MD, Williams K, Kuntz G, Umscheid CA: **When the decision is what to decide: using evidence inventory reports to focus health technology assessments.** *International Journal of Technology Assessment in Health* 2011, **27:**127-132.

26. Moat KA, Lavis JN, Abelson J: **How contexts and issues influence the use of policy-relevant research syntheses: a critical interpretive synthesis.** *Milbank Q* 2013, **91:**604-648.

27. Mossialos E, Naci H, Courtin E: **Expanding the role of community pharmacists: policymaking in the absence of policy-relevant evidence?** *Health Policy* 2013, **111:**135-148.

28. Munn Z, Lockwood C, Moola S: **The Development and Use of Evidence Summaries for Point of Care Information Systems: A Streamlined Rapid Review Approach.** *Worldviews Evid Based Nurs* 2015, **12:**131-138.

29. Murthy L, Shepperd S, Clarke MJ, Garner SE, Lavis JN, Perrier L, Roberts NW, Straus SE: **Interventions to improve the use of systematic reviews in decision-making by health system managers, policy makers and clinicians.** *Cochrane Database Syst Rev* 2012, **9:**CD009401.

30. Nannini A, Houde SC: **Translating evidence from systematic reviews for policy makers.** *J Gerontol Nurs* 2010, **36:**22-26.

31. Noor RA: **Health research oversight in Africa.** *Acta Trop* 2009, **112 Suppl 1:**S63-70.

32. Nutley T, Gnassou L, Traore M, Bosso AE, Mullen S: **Moving data off the shelf and into action: an intervention to improve data-informed decision making in Cote d'Ivoire.** *Glob Health Action* 2014, **7:**25035.

33. Oermann MH, Roop JC, Nordstrom CK, Galvin EA, Floyd JA: **Effectiveness of an intervention for disseminating Cochrane Reviews to nurses.** *Urol Nurs* 2009, **29:**35-39, 54.

34. Oliver K, Innvar S, Lorenc T, Woodman J, Thomas J: **A systematic review of barriers to and facilitators of the use of evidence by policymakers.** *BMC Health Serv Res* 2014, **14:**2.

35. Perrier L, Mrklas K, Lavis JN, Straus SE: **Interventions encouraging the use of systematic reviews by health policymakers and managers: a systematic review.** *Implement Sci* 2011, **6:**43.

36. Perrier L, Mrklas K, Shepperd S, Dobbins M, McKibbon KA, Straus SE: **Interventions encouraging the use of systematic reviews in clinical decision-making: a systematic review.** *J Gen Intern Med* 2011, **26:**419-426.

37. Perrier L, Persaud N, Thorpe KE, Straus SE: **Using a systematic review in clinical decision making: a pilot parallel, randomized controlled trial.** *Implement Sci* 2015, **10:**118.

38. Rosenbaum SE, Glenton C, Wiysonge CS, Abalos E, Mignini L, Young T, Althabe F, Ciapponi A, Marti SG, Meng Q, et al: **Evidence summaries tailored to health policy-makers in low- and middle-income countries.** *Bull World Health Organ* 2011, **89:**54-61.

39. Santesso N, Rader T, Nilsen ES, Glenton C, Rosenbaum S, Ciapponi A, Moja L, Pardo JP, Zhou Q, Schunemann HJ: **A summary to communicate evidence from systematic reviews to the public improved understanding and accessibility of information: a randomized controlled trial.** *J Clin Epidemiol* 2015, **68:**182-190.

40. Sullivan SM, Coyle D, Wells G: **What guidance are researchers given on how to present network meta-analyses to end-users such as policymakers and clinicians? A systematic review.** *PLoS One* 2014, **9:**e113277.

41. Taylor-Robinson D, Milton B, Lloyd-Williams F, O'Flaherty M, Capewell S: **Policy-makers' attitudes to decision support models for coronary heart disease: a qualitative study.** *J Health Serv Res Policy* 2008, **13:**209-214.

42. Thomson H: **Improving utility of evidence synthesis for healthy public policy: the three Rs (relevance, rigor, and readability [and resources]).** *Am J Public Health* 2013, **103:**e17-23.

43. Thomson HJ, Thomas S: **The effect direction plot: visual display of non-standardised effects across multiple outcome domains.** *Res Synth Methods* 2013, **4:**95-101.

44. Tricco AC, Cardoso R, Thomas SM, Motiwala S, Sullivan S, Kealey MR, Hemmelgarn B, Ouimet M, Hillmer MP, Perrier L, et al: **Barriers and facilitators to uptake of systematic reviews by policy makers and health care managers: a scoping review.** *Implement Sci* 2016, **11:**4.

45. Wallace J, Byrne C, Clarke M: **Making evidence more wanted: a systematic review of facilitators to enhance the uptake of evidence from systematic reviews and meta-analyses.** *Int J Evid Based Healthc* 2012, **10:**338-346.

46. Wilson MG, Moat KA, Lavis JN: **The global stock of research evidence relevant to health systems policymaking.** *Health Res Policy Syst* 2013, **11:**32.

47. Yavchitz A, Ravaud P, Hopewell S, Baron G, Boutron I: **Impact of adding a limitations section to abstracts of systematic reviews on readers' interpretation: a randomized controlled trial.** *BMC Med Res Methodol* 2014, **14:**123.
